# Association of the *WNT3* polymorphisms and non-syndromic cleft lip with or without cleft palate: evidence from a meta-analysis

**DOI:** 10.1042/BSR20181676

**Published:** 2018-11-23

**Authors:** Bing-qian Wang, Shu-tao Gao, Kun Chen, Zhu-qiu Xu, Jia-ming Sun, Yun Xia, Zheng-tao Lv

**Affiliations:** 1 Department of Plastic Surgery, Union Hospital, Tongji Medical College, Huazhong University of Science and Technology, Wuhan 430022, China; 2 Department of Spine Surgery, The First Affiliated Hospital of Xinjiang Medical University, Urumqi, Xinjiang 830054, China; 3 Department of Orthopedics, Tongji Hospital, Tongji Medical College, Huazhong University of Science and Technology, Wuhan 430030, China; 4 Plastic Surgery Hospital, Chinese Academy of Medical Sciences, Peking Union Medical College, Beijing 100730, China

**Keywords:** meta analysis, non-syndromic cleft lip with or without cleft palate, polymorphism, wnt3

## Abstract

Objective: This meta-analysis was conducted with the aim of investigating the association between *WNT3* gene polymorphisms and non-syndromic cleft lip (CL) with or without cleft palate (NSCL/P) predisposition. Methods: A comprehensive literature search was performed in six online databases including PubMed, Embase, ISI Web of Science, CENTRAL, CNKI, and Wanfang from inception up to June 2018 without language restriction. Pooled odds ratios (ORs) and corresponding 95% confidence intervals (95%CIs) were calculated under allele model of inheritance to indicate the association between *WNT3* polymorphisms and NSCL/P. Risk of bias was assessed through the Newcastle–Ottawa scale (NOS). Predetermined stratified and sensitivity analyses were performed using the RevMan 5.3 software, publication bias were evaluated by Egger’s and Begg’s tests. Results: Seven case–control studies comprising 1617 NSCL/P patients and 2143 healthy controls were identified and included in the present study, a total of eight loci were investigated in the present study: rs3809857 was significantly associated with NSCL/P vulnerability (G compared with T, OR = 1.34, 95%CI: 1.15–1.56, *P*=0.0001), a significant association between rs9890413 polymorphism and NSCL/P susceptibility (A compared with G, OR = 1.25, 95%CI: 1.06–1.47, *P*=0.007) was detected as well. Since only few studies reported detailed data about the association between rs142167, rs7207916, rs199498, rs111769, rs12452064, rs11653738, and NSCL/P risk, these results were not combined using meta-analysis. Conclusion: Based on the findings of our current study, the rs3809857 and rs9890413 polymorphisms of *WNT3* appeared to be associated with NSCL/P. Limited evidence is found to support the association between other *WNT3* polymorphisms and risk of NSCL/P.

## Introduction

Non-syndromic cleft lip (CL) with or without cleft palate (NSCL/P) is one of the most frequent congenital malformation amongst live births worldwide. The global prevalence of NSCL/P ranging from 1:500 to 1:2500 live births, with considerable variability across geographic origin, ethnic and racial background, as well as socioeconomic status [1]. Based on clinical manifestation, NSCL/P can be generally divided into CL, cleft palate (CP), and CL and palate (CLP) [2]. Since NSCL/P affects speech, feed, as with appearance, individuals with NSCL/P suffer greatly from both functional and cosmetic problems, which may bring about delayed development, weak social integration, and poor quality of life. In addition, a long term follow-up study has reported that NSCL/P is significantly correlated with increased risk of overall and cause specific mortality [3]. Although advanced surgical treatment and rehabilitation have enabled sufferers to normal life, NSCL/P inevitably imposes a heavy burden on both family and society [4].

As a complex disorder, current knowledge about the etiopathogenesis of NSCL/P remains largely undetermined. Environmental variations, genetic influences and interactions of these factors all drive the occurrence of NSCL/P. Researchers have reported that exposure to environmental factors like drinking, tobacco smoking, medicinal drugs, viral infection, and lack of vitamins during early pregnancy appear to increase the risk of having NSCL/P offspring [5,6]. Apart from environmental factors, genetic factors are as well considered to participate in the pathogenesis of NSCL/P. Estimates of the risk of recurrence for first-degree relatives range from 24- to 82-fold compared with the general population [7]. The strong family aggregation suggests a large genetic component for NSCL/P. Across the past decade, mounting studies have reported a huge number of gene polymorphisms of various candidate genes including *IRF6* [8], *TGFA* [9], *MTHFD1* [10], *MTHFR* [11], *BMP4* [12], and so forth, that could be related to NSCL/P vulnerability.

The *WNT* gene family consists of a range of members such as *WNT3, WNT3A, WNT5A, WNT8A, WNT11*, and so forth [13]. The products of *WNT* genes constitute a cluster of conserved secreted glycoproteins that participant in developmental and cell-biological processes, thus playing a fundamental role in craniofacial embryogenesis [14,15]. Mutations in *WNT* genes and their downstream targets have been implicated in human NSCL/P [16]. Of the *WNT* family, *WNT3* gene is the most extensively studied candidate gene for NSCL/P.

Early in 2008, Chiquet et al. [17] initially reported the correlation between *WNT3* gene polymorphisms and NSCL/P vulnerability, and found some locus polymorphisms significantly associated with NSCL/P. Afterward, several replicate studies were performed across different races with inconsistent outcomes [18–24]. Respecting the sample size was small and the statistical power was limited of individual study, we conducted the present systematic review and meta-analysis to provide a more comprehensive and precise estimation on the association between *WNT3* polymorphisms and NSCL/P predisposition.

## Materials and methods

The current systematic review and meta-analysis was performed in accordance with PRISMA (the Preferred Reporting Items for Systematic Review and Meta-Analyses) guidelines [25]. An unpublished protocol was prepared for internal comment.

### Literature search strategy

The computerized literature search of the present study was systematically conducted using six online electronic databases including PubMed, Embase, ISI Web of Science, CENTRAL, CNKI, and Wanfang. All the relevant literature was published previous to June 2018. A combination of Medical Subject Headings (MeSH) alongside free terms was utilized to hunt all the potentially eligible publications without any language restriction. For English databases, we employed the following search string: (Single Nucleotide Polymorphism or polymorphism or SNP or SNPs or ‘Polymorphism, Single Nucleotide’ [MeSh]), and (cleft lip or cleft palate or oral cleft or ‘Cleft Lip’ [MeSh] or ‘Cleft Palate’ [MeSh] or NSCL/P or NSCLP), and Wnt3. For Chinese academic databanks, we adopted the key words to identify related Chinese literature, the literature search strategy was as followed: ‘Chun E Lie’, ‘WNT3’, and ‘Duo Tai Xing’. The reference lists of relevant reviews and full-text articles were manually examined as well to look for additional possible studies.

### Inclusion and exclusion criteria

The PICOS principle was followed to establish the inclusion criteria for our current study. Studies satisfying the following criteria were included for review: (i) NSCL/P should be diagnosed on the basis of clinical examination; (ii) studies on the relationship between *WNT3* polymorphisms and NSCL/P susceptibility that have been published; (iii) control subjects should be defined as healthy subjects without history of cleft and other major congenital anomalies; (iv) available odds ratio (OR) and 95% confidence interval (95%CI) under allelic comparison of individual loci; (v) observational studies (case–control or cohort studies) on humans.

Accordingly, studies would be excluded if they met the following criteria: (i) animal studies, reviews, case reports, conference abstracts, as well as editorials; (ii) data that overlapped previous publications. If duplicated studies reporting overlapping data were identified, the most comprehensive one was included in the meta-analysis.

### Methodological quality assessment

The methodological quality of eligible studies was evaluated separately by the first two investigators according to the Newcastle–Ottawa scale (NOS) for observational studies [26]. A ‘star system’ was applied to judge individual study on three broad perspectives: the selection of the case and control groups; the comparability of the case and control groups, and the ascertainment of either the exposure or outcome of interest for case–control studies. Higher scores indicate better methodological quality of included studies. Two reviewers (B.-q.W. and S.-t.G.) independently assessed the risk of bias of each included study, the results were compared afterward. Disagreements between two investigators were settled by discussion until a mutual consensus was reached.

### Data extraction

In compliance with the predefined criteria, the following information was meticulously extracted independently by two reviewers (B.-q.W. and S.-t.G.) from all qualified articles: (i) surname of the first author; (ii) year of publication; (iii) variant locus; (iv) country where the study was performed; (v) ethnicity of enrolled subjects; (vi) numbers of case and control subjects; (vii) results of Hardy–Weinberg equilibrium (HWE) test; (viii) OR and 95%CI under allelic comparison of individual locus; (ix) major allele distribution of case and control participants. Once encountering discrepancy during this process, two authors re-inspected the article together and reached an agreement after discussion.

### Statistical analysis

The strength of association between *WNT3* polymorphisms and NSCL/P susceptibility was evaluated through merging ORs and corresponding 95%CIs of individual studies. The between-study heterogeneity was assessed using the *Q*-statistical test and *I^2^* test [27]. The random-effect model and fixed-effect model were used for data combination in the presence (*P*<0.1, *I^2^* > 50%) or absence of heterogeneity (*P*>0.1, *I^2^*< 50% indicates acceptable heterogeneity), respectively [28,29]. The leave-one-out sensitivity analysis was conducted by removing each study in turn and reassessing the resulting effect on the overall effect. Egger’s regression test and Begg’s rank correlation test were used to estimate the publication bias (Stata version 12.0, Stata Corp LP, U.S.A.) [30]. Forest plots and funnel plots were generated using RevMan 5.3 software (Copenhagen: The Nordic Cochrane Centre, The Cochrane Collaboration, 2014). In addition, stratified analyses were performed according to subtypes of NSCL/P (CL, CP, or CLP) and ethnicity of NSCL/P patients.

### Functional predictions

Two *in silico* tools were applied to predict the function of *WNT3* polymorphisms: HaploReg 4.1 [31] (http://pubs.broadinstitute.org/mammals/haploreg/haploreg.php) and GTEx Project [32] (https://gtexportal.org/home/).

## Results

### Literature search

**Figure 1:**
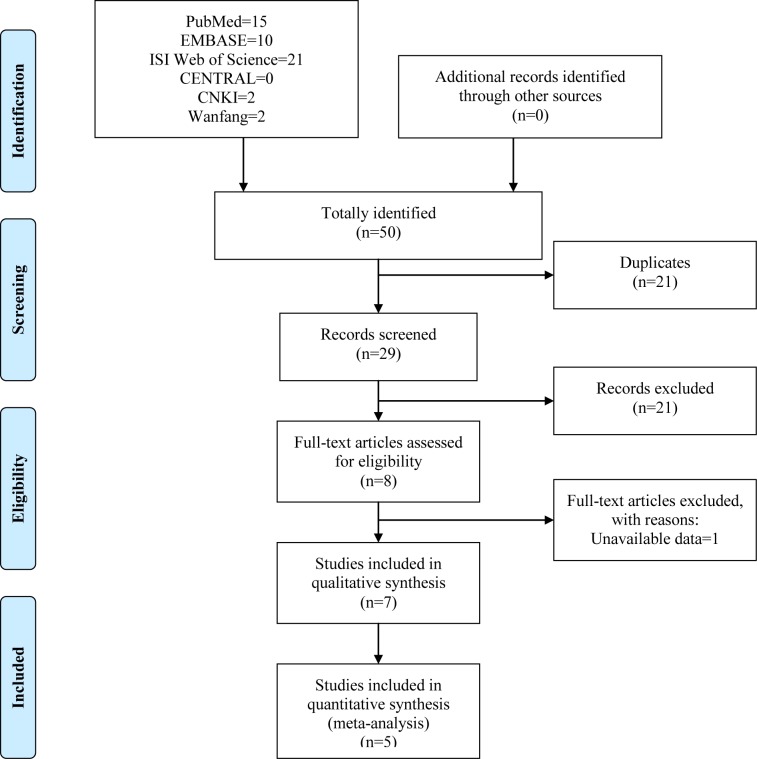
Flow diagram of literature search and screen

The initial search of six online databases yielded 50 records comprising 15 from PubMed, 10 from EMBASE, 21 from ISI Web of Science, 0 from CENTRAL, 2 from CNKI, and 2 from Wanfang. No record was identified through other sources. Overall, a total of 50 records were identified. After the first-stage scanning of title and author, 21 duplicated records were excluded. Of the remaining 29 records, a further 21 citations were eliminated after title and abstract screening. The remaining eight articles went into full-text assessment for eligibility, and one was removed because of unavailable data [17]. Seven studies [18–24] were incorporated into the qualitative synthesis, and five [19,21–24] were included in the final meta-analysis. The process of literature selection process is presented in Figure 1.

### Main characteristics

The general characteristics of included studies were presented in Table 1. Seven case–control studies comprising 1617 NSCL/P patients and 2143 healthy controls were identified and included in the present study. One of the studies [18] was published in Chinese and the rest were published in English. The included researches were published between 2010 and 2018. All the studies were case–control studies in design. Of them, two were carried out in China [18,23], two in Iran [19,24], one in U.S.A. [22], one in Poland [21], and another one in Estonia [20]. The sample size of individual study ranged from 104 to 463 for cases, and 112 to 606 for controls. A total of eight loci were investigated in the present study, including four studies on rs3809857, five on rs9890413, two on rs142167, two on rs7207916, one on rs199498, one on rs111769, one on rs12452064, and one on rs11653738. None of the included studies deviated from the HWE with the exception of Farrokhi Karibozorg et al. [24] study for rs3809857 polymorphism. On the basis of the NOS, each study received no less than five stars for methodological quality assessment, as shown in Table 2.

**Table 1:** Main characteristics of included studies

Study	Country	Ethnicity	Variant	NSCL/P / control	Major allele (%)		HWE	Association		
					NSCL/P	Control		OR	95%CI	*P*-value
Farrokhi Karibozorg (2018) [24]	Iran	Asian	*rs3809857 (G/T)*	113/220	63.7	57.1	<0.01	1.32	0.95–1.83	0.1
			*rs9890413 (A/G)*	113/220	78.8	78.9	0.89	0.94	0.64–1.39	0.77
Lu (2015) [23]	China	Asian	*rs3809857 (G/T)*	236/400	76.5	69.9	0.05	1.4	1.08–1.82	0.01
			*rs9890413 (A/G)*	236/400	92.6	96.9	0.60	0.4	0.24–0.68	0.0007
Menezes (2010) [22]	U.S.A.	Caucasian	*rs142167 (A/G)*	463/303	NA	NA	>0.05	1.61	1.29–2.02	2.80E-05
			*rs199498 (C/T)*	463/303	NA	NA	>0.05	1.34	1.06–1.68	0.01
			*rs111769 (C/T)*	463/303	NA	NA	>0.05	0.73	0.59–0.90	0.003
			*rs9890413 (A/G)*	463/303	NA	NA	>0.05	1.40	1.12–1.74	0.002
Mostowska (2012) [21]	Poland	Caucasian	*rs3809857 (G/T)*	210/244	63.8	57.8	0.81	1.29	0.99–1.68	0.06
			*rs12452064 (A/G)*	210/244	55.7	55.1	0.41	1.02	0.79–1.33	0.86
			*rs7207916 (A/G)*	210/244	59.0	62.5	0.90	0.87	0.66–1.13	0.29
			*rs9890413 (A/G)*	210/244	73.3	69.3	0.83	1.22	0.91–1.63	0.18
Nikopensius (2010) [20]	Estonia	Caucasian	*rs11653738 (T/C)*	104/606	41.4	31.7	>0.05	1.518	1.123–2.053	0.0064
Rafighdoost (2018) [19]	Iran	Asian	*rs3809857 (G/T)*	120/112	76.7	71.0	0.77	1.34	0.89–2.04	0.16
			*rs9890413 (A/G)*	120/112	90.8	88.8	0.05	1.24	0.68–2.28	0.48
Xin (2013) [18]	China	Asian	*rs142167 (G/A)*	371/258	66.2	69.0	0.82	0.88	0.69–1.12	0.29
			*rs7216231 (A/G)*	371/258	64.7	68.2	0.86	0.85	0.67–1.08	0.19

Abbreviations: NA, not available.

**Table 2:** Quality assessment of included studies

Item/study	Farrokhi Karibozorg (2018)	Lu (2015)	Menezes (2010)	Mostowska (2012)	Nikopensius (2010)	Rafighdoost (2018)	Xin (2013)
Adequate definition of cases	*	*	*	*	*	*	*
Representativeness of cases	-	-	-	-	-	-	-
Selection of control subjects	-	-	-	-	*	-	-
Definition of control subjects	*	*	*	*	*	*	*
Control for important factor or additional factor	*	-	-	**	-	**	-
Exposure assessment	*	*	*	*	*	*	*
Same method of ascertainment for all subjects	*	*	*	*	*	*	*
Non-response rate	*	*	*	*	*	*	*

A study could be awarded a maximum of one star for each item except for the item ‘Control for important factor or additional factor’.

The definition/explanation of each column of the NOS is available from http://www.ohri.ca/programs/clinical_epidemiology/oxford.asp.

### Quantitative data analyses

Quantitative data analysis was planned to be performed for each locus polymorphism in the *WNT3* gene. However, owing to limited degrees of freedom, meta-analyses were only carried out on rs3809857 and rs9890413.

#### rs3809857 polymorphism and NSCL/P susceptibility

**Figure 2:**
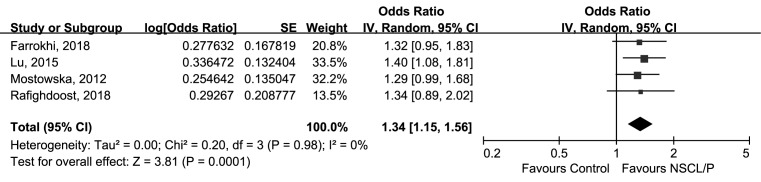
Forest plot of rs3809857 in *WNT3* gene and risk of NSCL/P

Meta-analysis for rs3809857 polymorphism and NSCL/P susceptibility was available on four studies including 679 cases and 976 controls [19,21,23,24]. As no heterogeneity amongst the four studies was observed (*P*=0.98, *I^2^* = 0%), the fixed-effect model was employed for calculating the association between rs3809857 and NSCL/P. The finding indicated that rs3809857 polymorphism was significantly associated with NSCL/P vulnerability (G compared with T, OR = 1.34, 95%CI: 1.15–1.56, *P*=0.0001, Figure 2).

#### rs9890413 polymorphism and NSCL/P susceptibility

**Figure 3:**
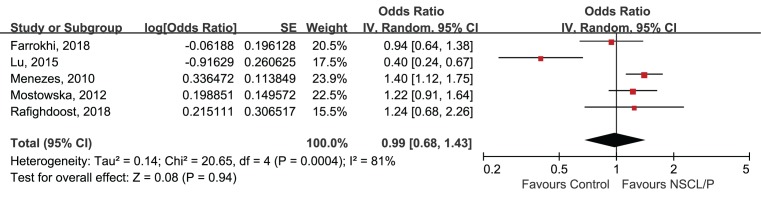
Forest plot of rs9890413 in *WNT3* gene and risk of NSCL/P

Meta-analysis for rs9890413 polymorphism and NSCL/P susceptibility was based on five studies [19,21–24], containing 1142 cases and 1279 controls. Because considerable heterogeneity amongst the five studies was detected (*P*=0.0004, *I^2^* = 81%), the random-effect model was applied for data combination. The result suggested a null association between rs9890413 polymorphism and NSCL/P vulnerability (A compared with G, OR = 0.99, 95%CI: 0.68–1.43, *P*=0.0004, Figure 3).

#### Other polymorphisms and NSCL/P susceptibility

Since only few studies reported detailed data about the association between rs142167, rs7207916, rs199498, rs111769, rs12452064, rs11653738, and NSCL/P risk, these results were not merged using a meta-analytic approach, providing a narrative description as an alternative.

Two studies reported rs142167 polymorphism and NSCL/P susceptibility with similar sample size but conflicting outcomes. Menezes et al. [22] found rs142167 was significantly associated with NSCL/P across Caucasian population (A compared with G, OR = 1.61, 95%CI: 1.29–2.02, *P*=0.000028), while the study by Xin et al. [18] found a null association between rs142167 and NSCL/P amongst Chinese population (OR = 0.88, 95%CI: 0.69–1.12, *P*=0.29).

Only one study investigated rs7207916 polymorphism and NSCL/P susceptibility [21]. No significant correlation was found between rs7207916 and NSCL/P amongst Polish population (A compared with G, OR = 0.87, 95%CI: 0.66–1.13; *P*=0.29).

Menezes et al. [22] reported the association of rs199498 as with rs111769 polymorphism and risk of NSCL/P. The two locus polymorphisms significantly associated with NSCL/P. rs199498 polymorphism appeared to increase NSCL/P risk (C compared with T, OR = 1.34, 95%CI: 1.06–1.68, *P*=0.01), yet rs111769 polymorphism tended to reduce NSCL/P risk (C compared with T, OR = 0.73, 95%CI: 0.59–0.90, *P*=0.003).

Mostowska et al. [21] studied the rs12452064 polymorphism and NSCL/P susceptibility. The major allele had a similar distribution in both cases and controls, and no significant relationship was observed between rs12452064 and NSCL/P (A compared with G, OR = 1.02, 95%CI: 0.79–1.33, *P*=0.86).

Nikopensius et al. [20] investigated the association of rs11653738 and NSCL/P susceptibility with 106 cases and 696 controls of Caucasian. Their findings suggested rs11653738 tended to significantly increase NSCL/P risk (T compared with C, OR = 1.518, 95%CI: 1.123–2.053, *P*=0.0064).

### Sensitivity analysis and publication bias

**Figure 4:**
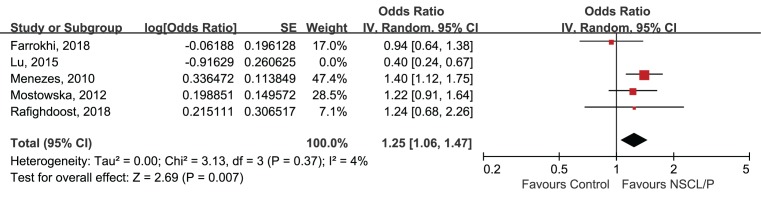
The leave-one-out sensitivity analysis of rs9890413 in *WNT3* gene and risk of NSCL/P

**Figure 5:**
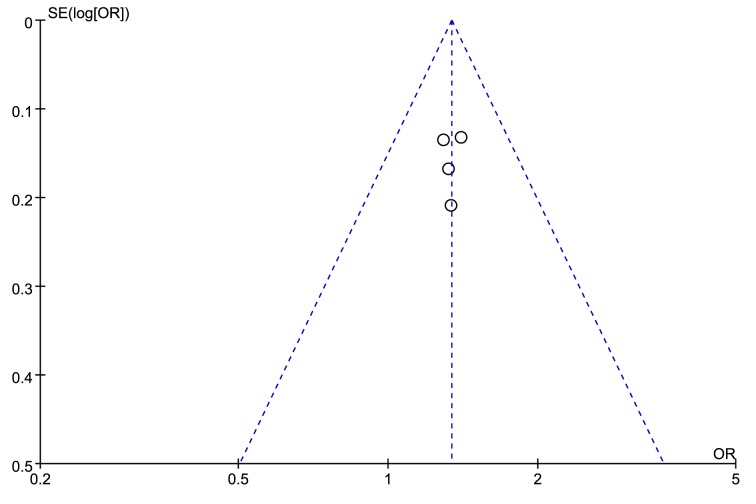
Funnel plot of rs3809857 in *WNT3* gene and risk of NSCL/P

**Figure 6:**
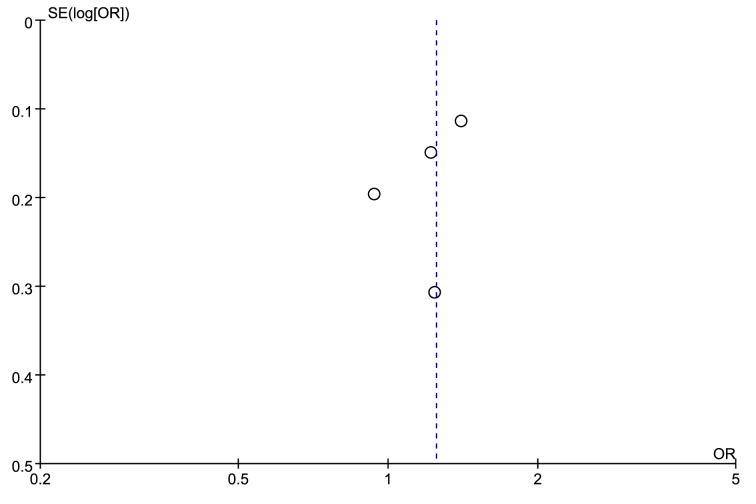
Funnel plot of rs9890413 in *WNT3* gene and risk of NSCL/P

The leave-one-out sensitivity analyses were employed to test the robustness and reliability of the outcomes. For rs9890413, when the study by Lu et al. [23] was removed, the overall effect changed and the heterogeneity became pretty small (*I^2^* = 4%, *P*=0.37). Therefore, this study was deemed as the source of heterogeneity across included studies, as a result we excluded this study from the meta-analysis and recalculated the OR and 95%CI for the association of rs9890413 and NSCL/P. The outcome became stable when performing sensitivity analysis again, and indicated a significant association between rs9890413 polymorphism and NSCL/P susceptibility (A compared with G, OR = 1.25, 95%CI: 1.06–1.47, *P*=0.007, Figure 4). Sensitivity analysis for rs3809857 polymorphism suggested a robust and credible result (detailed data not shown). The funnel plots were visually symmetrical (Figures 5 and 6). The Egger’s test (rs3809857: t = −0.21, *P*=0.856; rs9890413: t = −1.03, *P*=0.413) and Begg’s test (rs3809857: z = −0.34, *P*=1.000; rs9890413: z = 0.34, *P*=0.734) for rs3809857 and rs9890413 also suggested no statistically significant publication bias. Subgroup analyses by type of NSCL/P and ethnicity were not conducted in the present study because of limited number of included studies.

### Functional predictions

**Figure 7:**
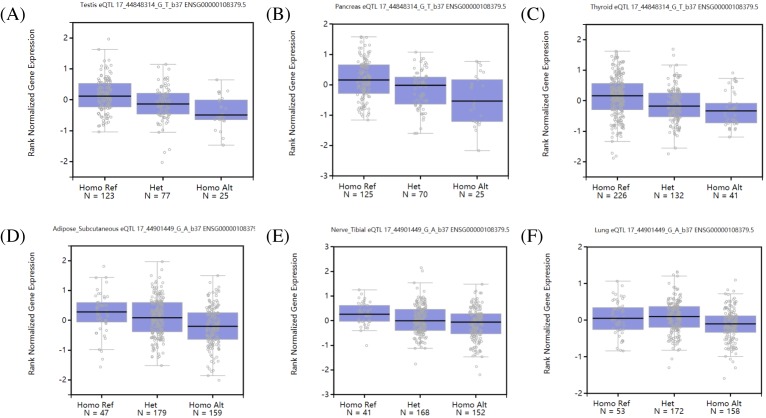
QTL box plots of rs3809857 and rs9890413 in different types of human tissues based on the GTEx analysis. eQTL box plots of rs3809857 and rs9890413 in human tissues: (**A**) rs3809857 and testis; (**B**) rs3809857 and pancreas; (**C**) rs3809857 and thyroid; (**D**) rs9890413 and subcutaneous adipose; (**E**) rs9890413 and tibial nerve; (**F**) rs9890413 and lung. Abbreviation: eQTL, expression Quantitative Trait Loci.

**Figure 8:**
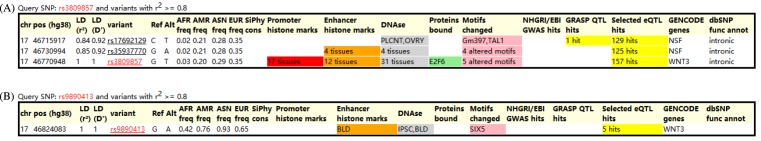
HaploReg view of rs3809857 and rs9890413 in WNT3 gene using HaploReg version 4.1. HaploReg view of rs3809857 and rs9890413: (**A**) rs3809857; (**B**) rs9890413.

The GTEx database was used to study the relationship between genetic variations and gene expressions in human tissue. We found that the T allele of rs3809857 is associated with lower expression in testis (*P*=1.8e-7; Figure 7A), pancreas (*P*=3.3e-7; Figure 7B), and thyroid (*P*=4.6e-7; Figure 7C); the G allele of rs9890413 is associated with lower WNT3 expression in subcutaneous adipose (*P*=0.0000024; Figure 7D), tibial nerve (*P*=0.000053; Figure 7E), and lung (*P*=0.000085; Figure 7F). HaploReg is a tool for exploring annotations of the non-coding genome based on well-established GWAS or novel datasets of variants, the results were shown in Figure 8.

## Discussion

Albeit recognition of risk factors for NSCL/P can help identify high-risk individuals, provide information for genetic consultation, as well as promote the establishment of preventive strategy, the precise pathophysiology of this disorder is not yet well understood. Intrinsic factors in synergy with extrinsic factors are proposed to be the potential etiology [33]. As an intrinsic factor, *WNT3* gene has received considerable attention over recent years. Several studies across different populations have been performed on the associations between *WNT3* polymorphisms and NSCL/P vulnerability.

However, there seems to have a race to discover genetic factors that are responsible for NSCL/P. Once finding a novel factor, there remains minimal interest to investigate it further, with most researchers aiming to look for new factors. The research on *WNT3* polymorphisms is without exception. Several polymorphisms in *WNT3* gene have been described, but only two of them were further studied by several authors and therefore appropriated for the final meta-analyses. Candidate gene association studies most commonly test for a difference in the allele frequency, the allele distributions in cases and controls could provide useful, direct summary statistics for the data. When several genetic model of inheritance are used for meta-analysis, a correction for multiple testing should be applied to avoid yielding false-positive results. Since insufficient information on genotype distribution was provided by our included studies, only allele effect was estimated in the current meta-analysis.

Majority of included studies presented insignificant correlation between rs3809857 and rs9890413 polymorphisms and NSCL/P vulnerability. Nevertheless, the pooled data indicated significant outcomes, which suggested restricted statistical power of each individual study. For rs9890413, when the study by Lu et al. was removed, the overall effect changed and the heterogeneity became insignificant (*I^2^* = 4%, *P*=0.37). In comparison with other included studies, Lu et al. reported a much higher A allele frequency in both NSCL/P group (Lu: 92.6% compared with mean: 81.0%) and control groups (Lu: 96.9% compared with mean: 79.0%). As a result, study by Lu et al. [23] was excluded in our meta-analysis. Variations in single nucleotide polymorphism (SNP) frequencies are seen across the major population groups because of random drift, novel mutations, and selection. It is possible that the A allele of rs9890413 plays a protective role against NSCL/P in Chinese, but it increases the risk of NSCL/P in the other populations, this hypothesis needs to be confirmed by further well-designed and population-based investigations with larger sample size.

Apart from the two loci, rs199498, rs111769, and rs11653738 polymorphisms appeared to significantly correlate to NSCL/P, but these significant findings were only supported by limited evidence, with only one or two studies focussing on these polymorphisms. A null association was yielded between rs7216231 and NSCL/P, but conflicting evidence was observed for the association of rs142167 and NSCL/P. Of included studies, three studies investigated whether there was subphenotype specificity between *WNT3* polymorphisms and NSCL/P. Only Menezes et al. [22] study with relatively large sample size obtained a positive result. Due to limited datasets of included studies, we were not allowed to carry out a precise subgroup analysis to determine etiological characteristics amongst CL, CP, as well as CLP.

The lip and palate in human embryo commences to differentiate at the fourth week and is completely formed by the twelfth week of gestation [34]. Formation of the upper lip and palate is a complex process and is composed of a range of highly co-ordinated steps during tissue morphogenesis, which are rigorously dominated by genetic networks. This process mainly comprises migration of ectoderm and formation of frontal, medial, and lateral nasal processes [34]. Malformation of the palate, lip, and nose are results of the disturbance of normal development.

It has been widely accepted that the *WNT* genes are widely implicated in regulating facial processes as well as upper lip fusion. Upon the binding of WNT ligands and Frizzled receptors, a series of intracellular signaling pathways are activated, which are well-known as canonical and non-canonical WNT pathways. *WNT* product is observed in the upper lip as well as primary and secondary palates [14]. *WNT3*, a vital member of *WNT* family, is mapped to chromosome 17q21.31-q21.32 [35]. *WNT3* is required at the earliest stages of human craniofacial development. Juriloff et al. [36] found the clf1 locus mapped in cleft susceptible mice contained *WNT3* genes. Lewis et al. [37] reported that head development is sensitive to the level of WNT3 signaling, and reducing the dose of WNT3 could partially rescue the truncated head phenotype. Moreover, Song et al. [38] have suggested that WNT3 could control lip development by binding to LRP6, which is a main co-receptor required to initiate the canonical WNT signaling. A large consanguineous family study has revealed that homozygous nonsense mutations in *WNT3* gene can result in craniofacial defects [39]. Considering the aforementioned reasons, variants directly or indirectly affecting the expression or protein properties of WNT3 could be plausible candidates that contribute to phenotypic differences amongst populations.

As the most abundant type of genetic variant, SNPs contribute substantially to phenotypic differences amongst individuals. The past two decades have witnessed great advance in the mapping of loci for NSCL/P. Regarding research on *WNT3* polymorphisms and NSCL/P, Chiquet et al. [17] first investigated the correlation between *WNT3* polymorphisms and the risk of developing NSCL/P amongst Hispanic and European American. rs9890413 located in the upstream of *WNT3* gene and rs3809857 within intron region of *WNT3* may not have direct effect on properties of WNT3 protein, they might influence the splicing and stability of non-coding RNA and thereby influence the risk of NSCL/P. Since only a few studies have been performed to evaluate the impact of *WNT3* polymorphisms on NSCL/P, whether and how the polymorphisms of *WNT3* contributes to the risk of NSCL/P has not been confirmed by functional experiments. In order to gain insights into the underlying mechanisms, we employed GTExPortal to test if there is a direct association between different genotypes of *WNT3* polymorphisms and altered expression of WNT3 protein in human tissues. Based on the results of expression Quantitative Trait Loci (eQTL), G allele of rs3809857 is associated with higher WNT3 expression in human testis (Figure 7A), pancreas (Figure 7B), and thyroid (Figure 7C), while A allele of rs9890413 is related with higher expression of WNT3 in subcutaneous adipose (Figure 7D), tibial nerve (Figure 7E), and lung (Figure 7F). Furthermore, the HaploReg 4.1 online database was applied to develop mechanistic hypotheses of the impact of rs3809857 (Figure 8A) and rs9890413 (Figure 8B). According to HaploReg, enhancer histone marks for rs3809857 were found in 12 human tissues, whereas enhancer histone marks for rs9890413 was only found in human blood. Additionally, rs3809857 was in linkage disequilibrium with rs17692129 and rs35937770 using a threshold of r^2^ ≥ 0.8. Notably, rs17692129 and rs35937770 are both polymorphisms within intron of *NSF* gene. As far as we know, NSF is involved in intracellular protein transport, but the impact of *NSF* polymorphisms on NSCL/P has not yet been confirmed. Taken together, both rs3809857 and rs9890413 have regulatory motifs changed, and both the variants are associated with altered expression of WNT3 in human organs, which could be one of the reasons why these polymorphisms confer risk of NSCL/P. To test these hypotheses, more functional experiments are still needed.

As far as we have known, the present study is the first comprehensive meta-analysis of the potential association between the *WNT3* polymorphisms and the risk of NSCL/P vulnerability. To improve the interpretation of the results, some limitations of the present study should be considered. First of all, in terms of the sample size, individual studies used hundreds but not thousands of participants, thus lacking adequately statistic power to guarantee the association. Second, we only investigated the role of eight loci polymorphisms separately. However, like most complex disorders, NSCL/P is caused by multiple loci polymorphisms and a string of genes with synergetic effects. Interactions of these loci and genes might conceal or magnify the actual function of a single locus polymorphism. Third, only five articles in English and two in Chinese from six databases were retrieved for the meta-analysis, potential relevant articles published in other languages might have been skipped, which might incur inclusion criteria bias. Fourth, due to lack of available data, we only conducted meta-analysis under allele model. At last, as all the component studies were hospital-based, the selection bias of participants might be introduced, hence limiting the extrapolation to general population of the current findings.

## Conclusion

More evidence is needed to uncover the association between *WNT3* polymorphisms and NSCL/P risk, based on the results of our meta-analysis we did find some genetic variants in *WNT3* gene that possibly contribute to the risk of NSCL/P. The present study suggested that G allele of rs3809857and A allele of rs9890413 polymorphisms in *WNT3* were significantly associated with increased NSCL/P vulnerability. Both rs3809857 and rs9890413 have regulatory motifs changed, and both the variants are associated with altered expression of WNT3 in human organs, which could be one of the reasons why these polymorphisms confer risk of NSCL/P. Insufficient evidence is found to support the association between other *WNT3* polymorphism and risk of NSCL/P. Concerning limitations of the current study, our findings need further confirmation by well-designed and population-based investigations with larger sample size amongst more ethnicities.

## Abbreviations

CL: cleft lip
CLP: CL and palate
CP: cleft palate
HWE: Hardy–Weinberg equilibrium
NOS: Newcastle–Ottawa scale
NSCL/P: non-syndromic CL with or without cleft palate
OR: odds ratio
SNP: single nucleotide polymorphism
95%CI: 95% confidence interval

## References

[1] Dixon M.J., Marazita M.L., Beaty T.H. and Murray J.C. (2011) Cleft lip and palate: understanding genetic and environmental influences. Nat. Rev. Genet. 12, 167–178 10.1038/nrg2933 21331089 PMC3086810

[2] Jugessur A., Farlie P.G. and Kilpatrick N. (2009) The genetics of isolated orofacial clefts: from genotypes to subphenotypes. Oral Dis. 15, 437–453 10.1111/j.1601-0825.2009.01577.x 19583827

[3] Christensen K., Juel K., Herskind A.M. and Murray J.C. (2004) Long term follow up study of survival associated with cleft lip and palate at birth. BMJ 328, 1405 10.1136/bmj.38106.559120.7C 15145797 PMC421777

[4] Wehby G.L. and Cassell C.H. (2010) The impact of orofacial clefts on quality of life and healthcare use and costs. Oral Dis. 16, 3–10 10.1111/j.1601-0825.2009.01588.x 19656316 PMC2905869

[5] Jia Z.L., Shi B., Chen C.H., Shi J.Y., Wu J. and Xu X. (2011) Maternal malnutrition, environmental exposure during pregnancy and the risk of non-syndromic orofacial clefts. Oral Dis. 17, 584–589 10.1111/j.1601-0825.2011.01810.x 21535328

[6] Gunnerbeck A., Edstedt Bonamy A.K., Wikstrom A.K., Granath F., Wickstrom R. and Cnattingius S. (2014) Maternal snuff use and smoking and the risk of oral cleft malformations–a population-based cohort study. PLoS ONE 9, e84715 10.1371/journal.pone.0084715 24454740 PMC3893163

[7] Sivertsen A., Wilcox A.J., Skjaerven R. (2008) Familial risk of oral clefts by morphological type and severity: population based cohort study of first degree relatives. BMJ 336, 432–434 10.1136/bmj.39458.563611.AE 18250102 PMC2249683

[8] Xia Y., Hu B., Chen J., Zheng L. and Song J. (2017) Association between the IRF6 rs2235371 polymorphism and the risk of nonsyndromic cleft lip with or without cleft palate in Chinese Han populations: a meta-analysis. Arch. Oral Biol. 84, 161–168 10.1016/j.archoralbio.2017.09.032 29017114

[9] Yan C., Deng-Qi H., Li-Ya C., Mang Y. and Ke-Hu Y. (2018) Transforming growth factor alpha Taq I polymorphisms and nonsyndromic cleft lip and/or palate risk: a meta-analysis. Cleft Palate Craniofac. J. 55, 814–820 10.1597/16-008 28001102

[10] Zhao H., Zhang J., Zhang M. (2015) Is MTHFD1 polymorphism rs 2236225 (c.1958G>A) associated with the susceptibility of NSCL/P? A systematic review and meta-analysis. F1000Res 4, 142 26834978 10.12688/f1000research.6425.1 PMC4722688

[11] Pan X., Wang P., Yin X. (2015) Association between maternal MTHFR polymorphisms and nonsyndromic cleft lip with or without cleft palate in offspring, a meta-analysis based on 15 case-control studies. Int. J. Fertil. Steril. 8, 463–480 25780529 10.22074/ijfs.2015.4186 PMC4355933

[12] Hu Y.Y., Qin C.Q., Deng M.H., Niu Y.M. and Long X. (2015) Association between BMP4 rs17563 polymorphism and NSCL/P risk: a meta-analysis. Dis. Markers 2015, 763090 10.1155/2015/763090 25648829 PMC4306361

[13] Wiese K.E., Nusse R. and van Amerongen R., Wnt signalling: conquering complexity. Development 145, 2018 10.1242/dev.165902 29945986

[14] Brugmann S.A., Goodnough L.H., Gregorieff A. (2007) Wnt signaling mediates regional specification in the vertebrate face. Development 134, 3283–3295 10.1242/dev.005132 17699607

[15] Vendrell V., Summerhurst K., Sharpe J., Davidson D. and Murphy P. (2009) Gene expression analysis of canonical Wnt pathway transcriptional regulators during early morphogenesis of the facial region in the mouse embryo. Gene Expr. Patterns 9, 296–305 10.1016/j.gep.2009.03.001 19303461

[16] He F. and Chen Y. (2012) Wnt signaling in lip and palate development. Front. Oral Biol. 16, 81–90 10.1159/000337619 22759672

[17] Chiquet B.T., Blanton S.H., Burt A. (2008) Variation in WNT genes is associated with non-syndromic cleft lip with or without cleft palate. Hum. Mol. Genet. 17, 2212–2218 10.1093/hmg/ddn121 18413325 PMC2852032

[18] Xin Y.-H., Ma L.-J., Zhai K. (2013) Association between single nucleotide polymorphism in Wnt3 and nonsyndromic cleft lip with or without cleft palate in Hui and Han population of Ningxia autonomous region. West China J. Stomatol., 2013, issue 4 397–402 23991581

[19] Rafighdoost H., Hashemi M., Asadi H. and Bahari G. (2018) Association of single nucleotide polymorphisms in WNT genes with the risk of nonsyndromic cleft lip with or without cleft palate. Congenit. Anom. (Kyoto) 58, 130–135 10.1111/cga.12271 29356097

[20] Nikopensius T., Jagomagi T., Krjutskov K. (2010) Genetic variants in COL2A1, COL11A2, and IRF6 contribute risk to nonsyndromic cleft palate. Birth Defects Res. A Clin. Mol. Teratol. 88, 748–756 10.1002/bdra.20700 20672350

[21] Mostowska A., Hozyasz K.K., Biedziak B., Wojcicki P., Lianeri M. and Jagodzinski P.P. (2012) Genotype and haplotype analysis of WNT genes in non-syndromic cleft lip with or without cleft palate. Eur. J. Oral Sci. 120, 1–8 10.1111/j.1600-0722.2011.00938.x 22288914

[22] Menezes R., Letra A., Kim A.H. (2010) Studies with Wnt genes and nonsyndromic cleft lip and palate. Birth Defects Res. A Clin. Mol. Teratol. 88, 995–1000 10.1002/bdra.20720 20890934 PMC2991560

[23] Lu Y.P., Han W.T., Liu Q. (2015) Variations in WNT3 gene are associated with incidence of non-syndromic cleft lip with or without cleft palate in a northeast Chinese population. Genet. Mol. Res. 14, 12646–12653 10.4238/2015.October.19.8 26505415

[24] Farrokhi Karibozorg H., Masoudian N., Saliminejad K., Ebadifar A., Kamali K. and Khorram Khorshid H.R. (2018) Association of the WNT3 Variations and the risk of non-syndromic cleft lip and palate in a population of Iranian infants. Avicenna J. Med. Biotechnol. 10, 168–172 30090211 PMC6064000

[25] Moher D., Liberati A., Tetzlaff J., Altman D.G. and PRISMA Group (2009) Preferred reporting items for systematic reviews and meta-analyses: the PRISMA statement. PLoS Med. 6, e1000097 10.1371/journal.pmed.1000097 19621072 PMC2707599

[26] Wells G.A., Shea B., O’Connell D. (2017) The Newcastle-Ottawa Scale (NOS) for assessing the quality of nonrandomised studies in meta-analyses. http://www.ohri.ca/programs/clinical_epidemiology/oxford.asp

[27] Higgins J.P., Thompson S.G., Deeks J.J. and Altman D.G. (2003) Measuring inconsistency in meta-analyses. BMJ 327, 557–560 10.1136/bmj.327.7414.557 12958120 PMC192859

[28] Mantel N. and Haenszel W. (1959) Statistical aspects of the analysis of data from retrospective studies of disease. J. Natl. Cancer Inst. 22, 719–748 13655060

[29] DerSimonian R. and Laird N. (1986) Meta-analysis in clinical trials. Control. Clin. Trials 7, 177–188 10.1016/0197-2456(86)90046-2 3802833

[30] Egger M., Davey Smith G., Schneider M. and Minder C. (1997) Bias in meta-analysis detected by a simple, graphical test. BMJ 315, 629–634 10.1136/bmj.315.7109.629 9310563 PMC2127453

[31] Ward L.D. and Kellis M. (2016) HaploReg v4: systematic mining of putative causal variants, cell types, regulators and target genes for human complex traits and disease. Nucleic Acids Res. 44, D877–D881 10.1093/nar/gkv1340 26657631 PMC4702929

[32] GTex Consortium (2013) The Genotype-Tissue Expression (GTEx) project. Nat. Genet. 45, 580–585 10.1038/ng.2653 23715323 PMC4010069

[33] Vieira A.R. (2012) Genetic and environmental factors in human cleft lip and palate. Front. Oral Biol. 16, 19–31 10.1159/000337521 22759667

[34] Shkoukani M.A., Chen M. and Vong A. (2013) Cleft lip - a comprehensive review. Front. Pediatr. 1, 53 10.3389/fped.2013.00053 24400297 PMC3873527

[35] Roelink H., Wang J., Black D.M., Solomon E. and Nusse R. (1993) Molecular cloning and chromosomal localization to 17q21 of the human WNT3 gene. Genomics 17, 790–792 10.1006/geno.1993.1412 8244403

[36] Juriloff D.M., Harris M.J., Dewell S.L. (2005) Investigations of the genomic region that contains the clf1 mutation, a causal gene in multifactorial cleft lip and palate in mice. Birth Defects Res. A Clin. Mol. Teratol. 73, 103–113 10.1002/bdra.20106 15690355

[37] Lewis S.L., Khoo P.L., De Young R.A. (2008) Dkk1 and Wnt3 interact to control head morphogenesis in the mouse. Development 135, 1791–1801 10.1242/dev.018853 18403408

[38] Song L., Li Y., Wang K. (2009) Lrp6-mediated canonical Wnt signaling is required for lip formation and fusion. Development 136, 3161–3171 10.1242/dev.037440 19700620

[39] Niemann S., Zhao C., Pascu F. (2004) Homozygous WNT3 mutation causes tetra-amelia in a large consanguineous family. Am. J. Hum. Genet. 74, 558–563 10.1086/382196 14872406 PMC1182269

